# Self-Tuning Fully-Connected PID Neural Network System for Distributed Temperature Sensing and Control of Instrument with Multi-Modules

**DOI:** 10.3390/s16101709

**Published:** 2016-10-14

**Authors:** Zhen Zhang, Cheng Ma, Rong Zhu

**Affiliations:** Department of Precision Instrument, Tsinghua University, Beijing 100084, China; zhangz14@mails.tsinghua.edu.cn (Z.Z.); zr_gloria@mail.tsinghua.edu.cn (R.Z.)

**Keywords:** MIMO, self-tuning, temperature control, instrument, high reliability

## Abstract

High integration of multi-functional instruments raises a critical issue in temperature control that is challenging due to its spatial–temporal complexity. This paper presents a multi-input multi-output (MIMO) self-tuning temperature sensing and control system for efficiently modulating the temperature environment within a multi-module instrument. The smart system ensures that the internal temperature of the instrument converges to a target without the need of a system model, thus making the control robust. The system consists of a fully-connected proportional–integral–derivative (PID) neural network (FCPIDNN) and an on-line self-tuning module. The experimental results show that the presented system can effectively control the internal temperature under various mission scenarios, in particular, it is able to self-reconfigure upon actuator failure. The system provides a new scheme for a complex and time-variant MIMO control system which can be widely applied for the distributed measurement and control of the environment in instruments, integration electronics, and house constructions.

## 1. Introduction

Miniaturization, modularization, and multi-functionalization have become development trends in instrumentation [1,2,3,4]. For example, Christian et al. [5] introduced one of the smallest real-time polymerase chain reaction (PCR) systems with a size of 100 × 60 × 33 mm^3^ and a weight of approximately 90 g. Segyeong et al. [6] presented a portable microfluidic flow cytometer with dual detection ability of impedance and fluorescence that is 150 × 100 × 100 mm^3^ in size and weighs nearly 800 g. Roda et al. [7] designed a simple and versatile portable device based on chemiluminescence lensless imaging that can simultaneously perform different types of bioassays.

For the instruments with multi-modules, internal temperature control becomes an important issue, since the different functions are achieved by several modules integrated in one box, and the modules’ thermal characters vary. For instance, the typical PCR temperatures are approximately 90 °C in denaturation, 55 °C in annealing, and 72 °C in extension [1]. Thus, these are three different temperatures in one cycle. The loop-mediated isothermal amplification (LAMP) temperature is set to a fixed value of 55 °C [2]. However, general modules require the environment temperature to be constant and near to room temperature. This is a challenge, since the modules are shaped differently and placed very close to each other due to spatial constraints.

Distributed temperature control is a general problem in various fields. Song et al. [8] introduced a method using artificial neural networks to optimize the temperature control in data centers. Shen et al. [9] designed a novel decoupling control system for high-dimensional, multi-input, multi-output (MIMO) room temperature control. Pohjoranta et al. [10] presented a method to control the temperature difference over the solid oxide fuel cell stack based on the predicted model. Moon et al. [11] introduced a temperature control method for an ultrasupercritical once-through boiler–turbine system using MIMO dynamic matrix control technology.

The distributed MIMO temperature control problem is complex because of strong coupling and time-variance in the internal structure. The modeling method is a common decoupling method with the estimation of the action object. Li et al. [12] introduced a decoupling method in a double-level air flow dynamic vacuum system based on neural networks and prediction principle. Gil et al. [13] presented a constrained nonlinear adaptive model-based control framework applied to a distributed solar collector field. Shen et al. [14] presented the temperature uniformity control of large-scale vertical quench furnaces with a proportional–integral–derivative (PID) decoupling control system to eliminate the strong coupling effects of multi-heating zones. Although the modeling method is an effective approach to dealing with the coupling effects in some cases, it cannot meet all the demands in practical application. Furthermore, the control performances of this method usually depend on the accuracy of the developed model, which highly restricts the robustness of the system.

The proportional–integral–derivative neural network (PIDNN) controller is independent of system modeling, and it is suitable for fan speed control [15,16,17]. Lee et al. [15] presented a PIDNN controller for a server fan cooling system. Rossomando et al. [16] used an adaptive neural PID controller for mobile robots’ trajectory tracking control. Maraba et al. [17] introduced a PIDNN-based speed control method for an asynchronous motor. To control a MIMO coupling fan cooling system, PIDNN controllers were used separately and thus could not deal with the coordination among the fans, which degraded the effectiveness of the control.

In this paper, a novel MIMO temperature sensing and control system based on a fully-connected PID neural network (FCPIDNN) is developed for an integrated instrument with multi-modules. The system takes the advantages of a PIDNN controller and full-connection neural networks, and capably adjusts the internal temperature of the instrument independent of object modeling. It enables the achievement of a global optimization by self-tuning, and makes a reconfiguration under failure event. The effectiveness of the control methodology is demonstrated and validated experimentally.

## 2. Problem Model and Design of Self-Tuning FCPIDNN Temperature Sensing and Control System

### 2.1. Temperature Control Problem Formulation

Due to the complexity of the thermohydrodynamic operation, it is generally difficult to build an accurate model for an instrument system. Stafford et al. [18] have made a thorough study at flat plate heat transfer with axial fan flows. They used infrared thermography to quantify a two-dimensional profile of the heat transfer coefficient on a flat plate for a range of fan speeds and the distances from fan to plate. A relationship between the fan speed and heat transfer intensity was presented. Generally, when the other conditions were kept stable, the faster the fan speed, the higher the Nusselt number in their experiments. Thus, the convective heat transfer characteristics can be changed by adjusting the fans’ speeds. Grimes et al. [19] have researched air flow and heat transfer in fan-cooled electronic systems. In their works, we can see that the heat transfer processes situated in the inlet and exit air flow from the fan varies significantly. The fan exhaling flow is unsteady and swirling, and the Nusselt number is higher, which enhances the heat transfer. However, this method makes the internal air more disordered.

Based on the previous research, when the other influencing factors keep invariant, we can get the function relation of temperature and fan speed in the presented mockup as:

T*_i_* = f*_i_* (F_1_,…, F*_j_*,…, F_6_), *i* = 1,…, 6 (1)

where T*_i_* is the sensor’s temperature around module *i*, and F*_j_* is the speed of cooling fan *j*. That is, the temperature variation is infulenced by the fans’ speed. As mentioned above, due to the complexity of the structural configuraiton and the thermohydrodynamic coupling, the function f_i_ cannot be obtained exactly. We can convert Equation (1) to another form:

(2) $$T_{i}=\sum_{k=1}^{6}(a_{\mathrm{ik}}\cdot F_{k}),\;k=1,\ldots ,\;6$$

*a_ik_* = *a_ik_* (F_1_,…, F*_j_*,…, F_6_), *j* = 1,…, 6 (3)

where *a_ik_* is the coefficient of F*_k_*, which is decided by F*_j_* (*j* = 1,…, 6). So, we can get the matrix form of the function relation as:

(4) $$\left(\begin{array}{c} T_{1}\\ \vdots \\ T_{6} \end{array}\right)=\left(\begin{array}{ccc} a_{11} & \ldots & a_{16}\\ \vdots & \ddots & \vdots \\ a_{61} & \ldots & a_{66} \end{array}\right)\left(\begin{array}{c} F_{1}\\ \vdots \\ F_{6} \end{array}\right)=A\left(\begin{array}{c} F_{1}\\ \vdots \\ F_{6} \end{array}\right)$$

where A stands for the relation matrix.

### 2.2. MIMO Temperature Sensing and Control System

As mentioned above, the PIDNN controller does not rely on the object’s model. The structure of the PIDNN controller is a three-layer network whose hidden layer neurons’ activation functions work as a PID controller [15,16,17]. The improved FCPIDNN controller proposed in this paper takes a series of PIDNN controllers as basic controllers and adds a full connection layer between the basic controllers and the cooling fans.

**Figure 1:**
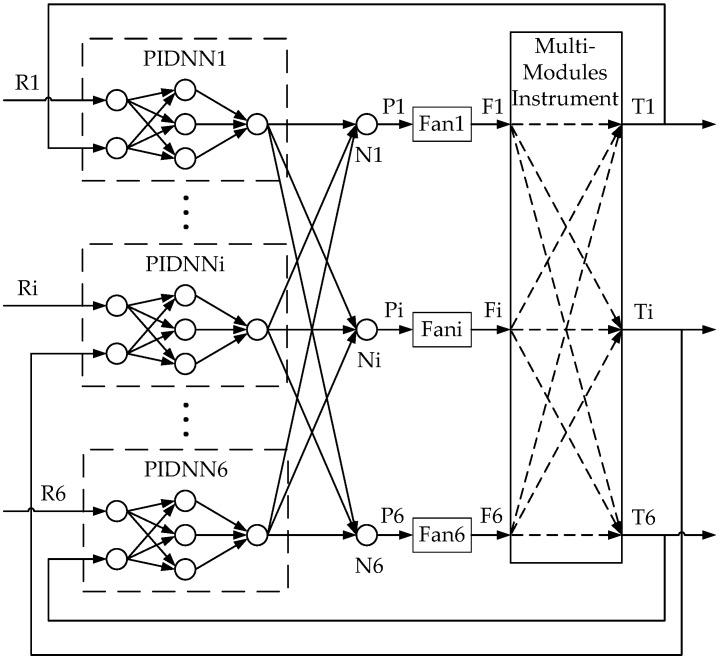
Structure of the fully-connected proportional–integral–derivative neural network (FCPIDNN) temperature controller for an instrument.

**Figure 2:**
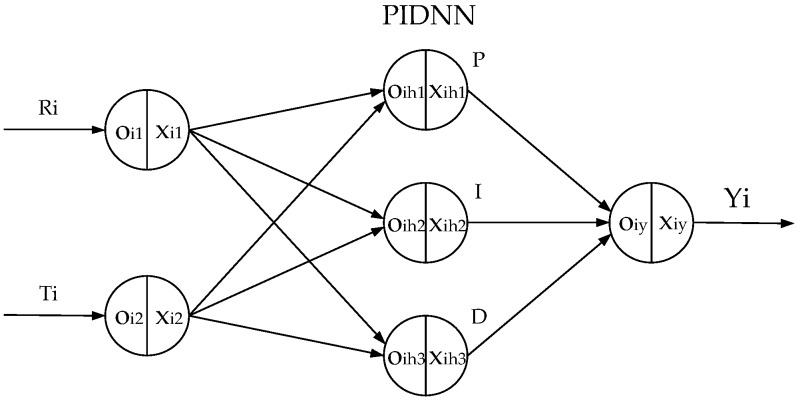
Structure of the PIDNN.

The structure of the FCPIDNN controller is shown in Figure 1. It contains a four-layer neural network. The symbol *i* represents the serial number (*i* = 1, ..., 6). R*_i_* is a target temperature. N*_i_* is the fourth layer neuron. P*_i_* is the pulse width modulation (PWM) control signal inputting to fan *i*, which is used to control fan speed. F*_i_* is the speed of fan *i*. T*_i_* is the local temperature of sensor *i*. The MIMO controller includes two main parts. One is six separate PIDNN controllers. The other is the full connection structure of the neural network. The structure of the PIDNN controller is shown in Figure 2, where R*_i_* is the target temperature. T*_i_* is the actual local temperature. Y*_i_* is the output of the PIDNN controller [15]. There are three layers in the PIDNN, including an input layer, a hidden layer, and an output layer. Each neuron has an input *o* and an output *x*. The input layer is used to estimate the difference between the set-point and the actual value. The hidden layer is composed of P, I, and D neurons, which implement a PID algorithm combined with connection weights from the hidden layer to the output layer. The output layer contains one neuron, summing the outputs of the hidden layer with weights. Here we utilize this kind of independent PIDNN mainly to reduce the complex calculating cost to achieve a real-time online training.

The full-connection structure from the output of the PIDNN to the fourth layer is mainly used to link the basic controllers to the fans. As seen in Equation (4), the relationship between the six fans and the output temperatures can be represented as a dimensional matrix. Under a slight change of the fan speed, this relationship can be approximately linear. Thereby, Equation (4) can be simplified as:

(5) $$\left(\begin{array}{c} T_{1}\\ \vdots \\ T_{6} \end{array}\right)=\left(\begin{array}{ccc} a_{11} & \ldots & a_{16}\\ \vdots & \ddots & \vdots \\ a_{61} & \ldots & a_{66} \end{array}\right)\left(\begin{array}{c} l_{1}P_{1}\\ \vdots \\ l_{6}P_{6} \end{array}\right)=\left(\begin{array}{ccc} a_{11} & \ldots & a_{16}\\ \vdots & \ddots & \vdots \\ a_{61} & \ldots & a_{66} \end{array}\right)\left(\begin{array}{ccc} l_{1}w_{o11} & \ldots & l_{1}w_{o61}\\ \vdots & \ddots & \vdots \\ l_{6}w_{o16} & \ldots & l_{6}w_{o66} \end{array}\right)\left(\begin{array}{c} Y_{1}\\ \vdots \\ Y_{6} \end{array}\right)$$

where $w_{\mathrm{oij}}$ is the weight from Y*_i_* to N*_j_*, and *l_i_* is the scale factor between P*_i_* to F*_i_*.

In the PIDNNi, the weights from the first layer to the second layer are *w_i_*_11_ = *w_i_*_12_ = *w_i_*_13_ = 1 and *w_i_*_21_ = *w_i_*_22_ = *w_i_*_23_ = −1, and the weight from the hidden layer to the output layer is *w_ihl_* (*l* = 1, ..., 3). The weight from the third layer to the fourth layer is *w_oij_*, where *i* and *j* present the serial number from PIDNN*i* to neuron N*j*. The goal of the FCPIDNN controller is to minimize the deviation of the actual temperature from the target temperature. The weights are updated online using the back-propagation (BP) algorithm. So, the cost function is defined as:

(6) $$J(n)=\;\frac{1}{2}\overset{6}{\underset{m=1}{\sum }}e_{m}^{2}(n)$$

where *e* represents the difference between the target and actual temperatures, *m* represents the number of temperature control channels, and *n* represents the sample number. The BP updated algorithm is used as:

(7) $$w_{oij}(n)=w_{oij}(n-1)-\eta_{o}\frac{\partial J(n)}{\partial w_{oij}(n)}$$

(8) $$w_{ihl}(n)=w_{ihl}(n-1)-\eta_{h}\frac{\partial J(n)}{\partial w_{ihl}(n)}$$

where $\eta_{o}$ and $\eta_{h}$ are the learning coefficients. The partial derivative terms in Equations (7) and (8) can be written as:

(9) $$\frac{\partial J}{\partial w_{oij}}=\overset{6}{\underset{m=1}{\sum }}\left(\frac{\partial J}{\partial e_{m}}\frac{\partial e_{m}}{\partial T_{m}}\frac{\partial T_{m}}{\partial F_{j}}\frac{\partial F_{j}}{\partial P_{j}}\frac{\partial P_{j}}{\partial w_{oij}}\right)$$

(10) $$\frac{\partial J}{\partial w_{ihl}}=\overset{6}{\underset{m=1}{\sum }}\left\{\frac{\partial J}{\partial e_{m}}\frac{\partial e_{m}}{\partial T_{m}}\left[\overset{6}{\underset{j=1}{\sum }}\left(\frac{\partial T_{m}}{\partial F_{j}}\frac{\partial F_{j}}{\partial P_{j}}\frac{\partial P_{j}}{\partial Y_{i}}\frac{\partial Y_{i}}{\partial w_{ihl}}\right)\right]\right\}$$

Due to the absence of the system model of the instrument, we use a sign function to deduce $\frac{\partial T_{m}}{\partial F_{j}}$ as:

(11) $$\frac{\partial T_{m}}{\partial F_{j}}=sgn\left[\frac{T_{m}(n)-T_{m}(n-1)}{F_{j}(n)-F_{j}(n-1)}\right]$$

### 2.3. Convergence Analysis

Based on Equation (5) to Equation (6) and Figure 2, the difference between J(n) and J(n−1) can be written as:

(12) $$△J(n)\;=\;J(n)\;-\;J(n\;-\;1)\;=\overset{6}{\underset{i=1}{\sum }}\overset{3}{\underset{l=1}{\sum }}\left(\frac{\partial J(n)}{\partial w_{ihl}(n)}△w_{ihl}(n)\right)+\overset{6}{\underset{i=1}{\sum }}\overset{6}{\underset{j=1}{\sum }}\left(\frac{\partial J(n)}{\partial w_{oij}(n)}△w_{oij}(n)\right)$$

According to Equation (7) to Equation (8), we can get

(13) $$△w_{oij}(n)\;=\;w_{oij}(n)\;-\;w_{oij}(n\;-\;1)\;=\;-\eta_{o}\frac{\partial J(n)}{\partial w_{oij}(n)}$$

(14) $$△w_{ihl}(n)\;=\;w_{ihl}(n)\;-\;w_{ihl}(n\;-\;1)\;=\;-\eta_{h}\frac{\partial J(n)}{\partial w_{ihl}(n)}$$

Introducing Equations (13) and (14) into Equation (12), we can get

(15) $$△J(n)\;=\;J(n)\;-\;J(n\;-\;1)=\overset{6}{\underset{i=1}{\sum }}\overset{3}{\underset{l=1}{\sum }}\left(-\eta_{h}\frac{\partial J(n)}{\partial w_{ihl}(n)}\frac{\partial J(n)}{\partial w_{ihl}(n)}\right)+\overset{6}{\underset{i=1}{\sum }}\overset{6}{\underset{j=1}{\sum }}\left(-\eta_{o}\frac{\partial J(n)}{\partial w_{oij}(n)}\frac{\partial J(n)}{\partial w_{oij}(n)}\right)$$

Equation (15) indicates that △J(n) ≤ 0, meaning that the cost function J(*n*) converges to the minimum, implying that the system is stable.

## 3. Experiments and Discussion

### 3.1. Instrument Mockup

**Figure 3:**
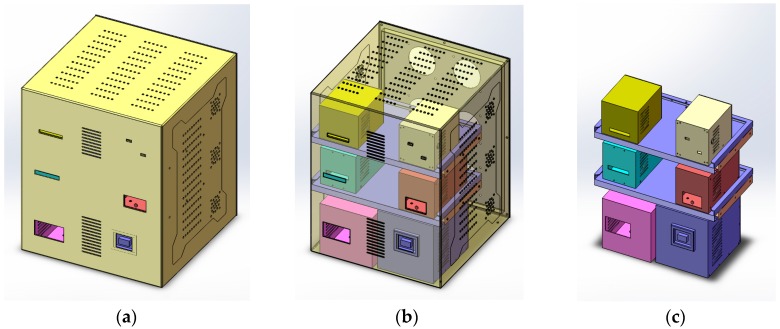
(**a**) The appearance of the instrument; (**b**) The perspective view of the 3D model with six modules inside and six cooling fans at the backplane; (**c**) The internal configuration of the instrument.

In practical applications, the different modules in an instrument have different power consumptions. To evaluate the proposed self-tuning FCPIDNN for temperature sensing and control, a mockup of a real instrument was constructed. The 3D model of the mockup is shown in Figure 3.

The mockup is 350 mm × 350 mm × 415 mm in volume with six modules inside. These modules have different shapes and volumes. Inside each module, there is a heater to generate heating and a small ventilation fan. The six heaters have different power, and the ventilation fans drive the heat out of the modules into internal public space of the mockup. Each module has a temperature sensor around itself, and there are six main cooling fans set at the back of the mockup. These cooling fans serve as temperature-tuning actuators to modulate the internal temperature of the instrument. Therefore, a temperature sensing and control system is built up with the modules, temperature sensors, and cooling fans.

### 3.2. Experimental System and Results

**Figure 4:**
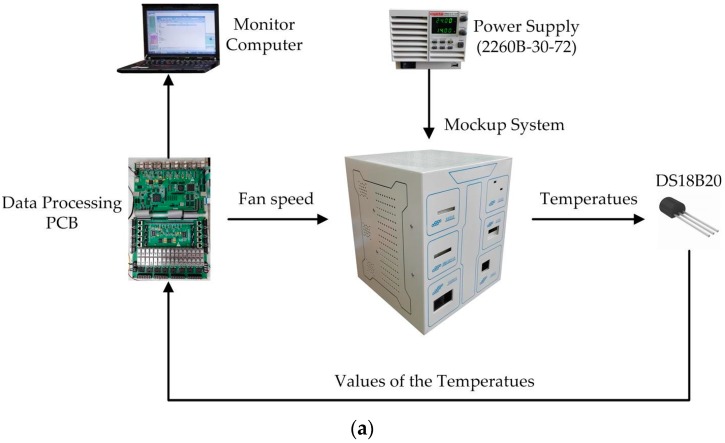

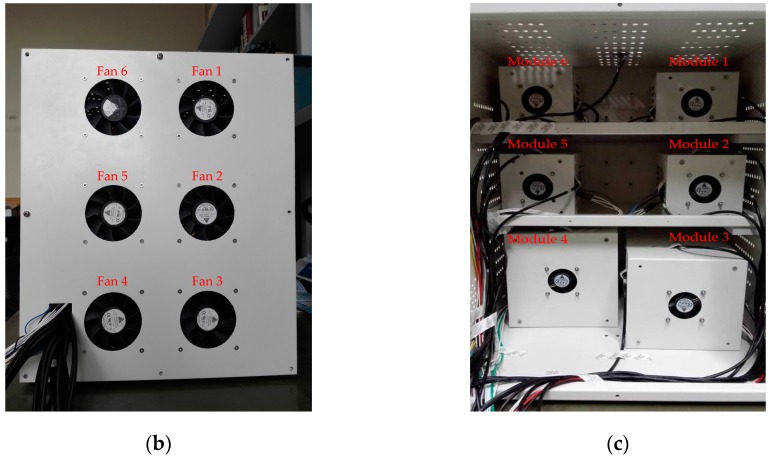
(**a**) Structure of the temperature sensing and control system; (**b**) Back view of the instrument mockup; (**c**) Internal layout of the mockup. PCB: printed circuit board.

The temperature sensing and control system was applied to a mockup of an instrument with multi-modules, as illustrated in Figure 4a. The instrumentation includes a mockup, a power supply (KEITHLEY 2260b-30-72), a temperature sensors (DS18B20) array, a tailor-designed data processing circuit, and a data monitoring computer. The system used the FCPIDNN controller mentioned above to perform the online temperature control. The details of the mockup have been presented previously. Figure 4b shows the back view of the mockup, with six fans indicated as fan1 to fan6. Figure 4c shows the internal layout of the mockup. The power supply was used to provide electrical energy to the heaters inside the six modules, simulating the heating behavior of the modules. The powers of the heaters inside modules 1 and 6, modules 2 and 3, and modules 4 and 5 are set to 24W, 48W, and 96W, respectively. The temperature sensors were used to detect the local temperatures around the six modules. The data processing circuit, including a field-programmable gate array (FPGA, EP2C35F484), acquired the temperatures around the six modules from temperature sensors through universal asynchronous receiver and transmitter (UART) port, implemented the developed self-tuning temperature control and outputted the PWM control signals to the fans. The data processing circuit also output the temperature information to the data monitoring computer through the UART port.

**Figure 5:**
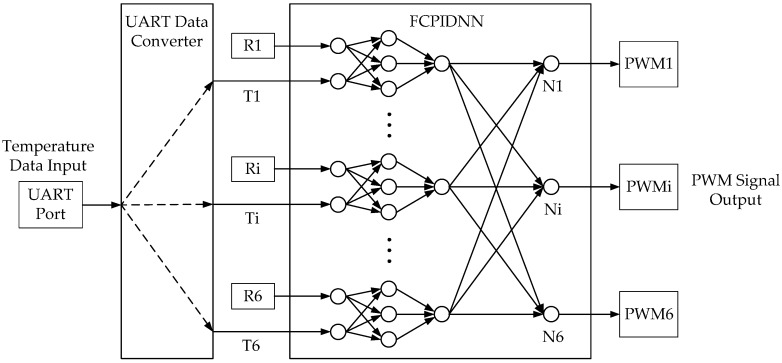
Structure of the FCPIDNN temperature controller for the instrument. PWM: pulse width modulation. UART: universal asynchronous receiver and transmitter.

Figure 5 shows the top level structure diagram of the FPGA’s program. The temperature signals are transported to the FPGA by a UART port. Then, the temperatures are extracted by a data converter and sent to the FCPIDNN controller together with the set-points. Finally, the calculated results are converted to the PWM signals and output to the fans. The number of the total logic elements used in the FPGA was 32,754. Specifically, the number of the total combination functions and dedicated logic registers are 32,020 and 4382, respectively.

To demonstrate the control performances of the proposed method in a MIMO temperature sensing and control system, we conducted three experiments and compared with the conventional method. The first experiment was conducted by comparing the temperature responses of the instrument by using a FCPIDNN controller and a traditional PIDNN controller. The second experiment was conducted to demonstrate the self-tuning of the controller when the target temperature changed in-process. The third experiment was conducted to simulate the reconfiguration of the control system when a pop-up actuator fail occurred.

**Figure 6:**
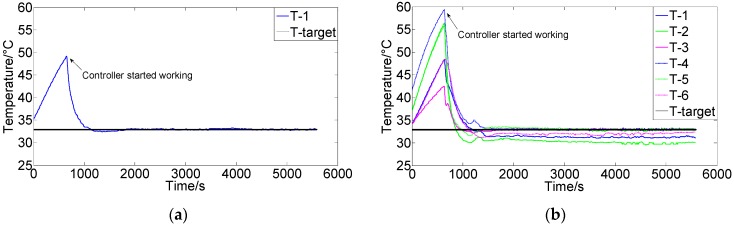
(**a**) Control results of a PIDNN controller for single module; (**b**) Control results of six separate PIDNN controllers for the six modules (Target temperature is 33 °C).

**Figure 7:**
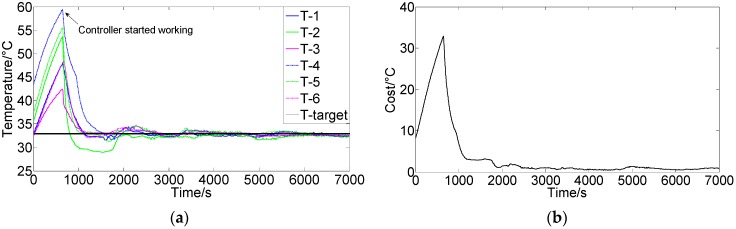
(**a**) Control results of the FCPIDNN controller (Target temperature 33 °C); (**b**) Variation of the square root of the cost function ($\sqrt{J(n)}$).

Figure 6 and Figure 7 show the control results using conventional PIDNN and FCPIDNN controllers, respectively. Figure 6a shows the results using a PIDNN controller to control a single module temperature. Figure 6b shows the results using six separate PIDNN controllers to control the temperatures of the six modules. Each controller worked in its own separate control loop. We can see from the results that the PIDNN could control the environmental temperature of one module very well, but brought steady-state errors when controlling for multiple modules. The steady-state temperatures of several controlled objects did not converge to the target, due to the presence of the mutual interference among the cooling fans. To solve this problem, we need to build connections among the controllers and thus ensure the controlling processes tending to global optimization.

Figure 7a shows the results using the FCPIDNN controller. The experimental condition was the same as that in Figure 6a. Figure 7b shows the corresponding variation of the square root of the cost function. We can see that the temperature of different modules tended to the setting targets simultaneously, and the cost function’s variation shows that the difference between the actual and target temperatures decreased and approached the minima. There were no steady-state errors in the temperature, and the control obtained a global convergence.

**Figure 8:**
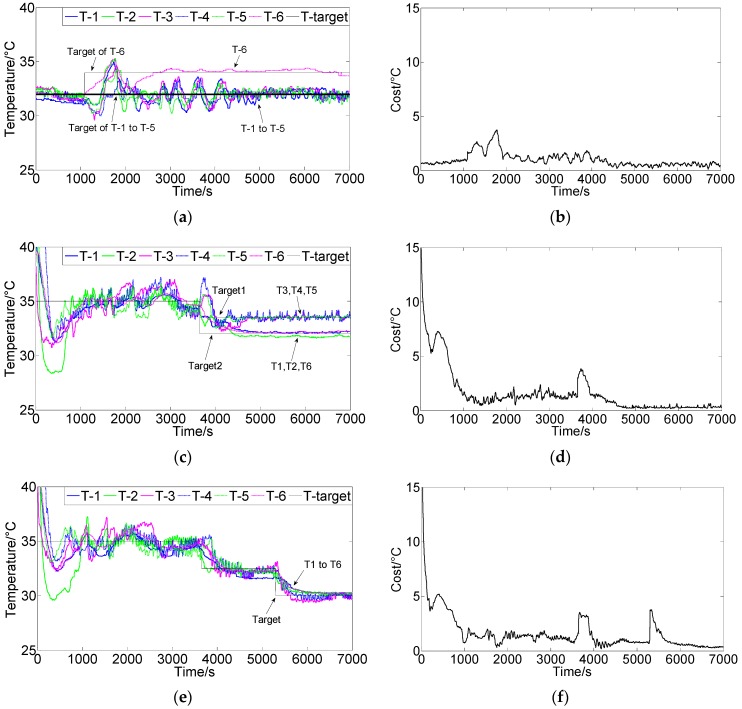
(**a**) Control results of the FCPIDNN controller when one temperature control target changed in a stable state; (**b**) Variation of the square root of the cost function ($\sqrt{J(n)}$) in process (a); (**c**) Control results of the FCPIDNN controller when temperature control targets changed to two different ones halfway; (**d**) Variation of the square root of the cost function ($\sqrt{J(n)}$) in process (c); (**e**) Control results of the FCPIDNN controller when temperature control targets changed twice in the control process; (**f**) Variation of the square root of the cost function ($\sqrt{J(n)}$) in process (e).

Figure 8 shows the control results using FCPIDNN controller when target temperature varied in process. There were three different control processes. Figure 8a,b (process 1) shows the control results when one temperature control target changed in a stable state. In the process, six controllers maintained the six modules’ temperature at 32 °C at the beginning. Then, the target temperature of module six was changed to 34 °C, and others’ temperatures remained unchanged. After a period of self-tuning by the FCPIDNN controller, the temperatures of all modules approached to their own targets, and the cost function gradually reduced to the minima. Figure 8c,d (process 2) show another control result when temperature target changed to different values. In the process, the temperature initially remained at 35 °C. Then, the target temperature for modules 1, 2, and 6 was changed to 33.5 °C, and the target temperature for modules 3, 4, and 5 was changed to 32 °C. After a period of self-tuning, all modules approached their target temperatures separately. Figure 8e,f (process 3) shows the control results when the target temperature for all modules was changed twice in the process; i.e., 35 °C at the beginning, then changed to 32.5 °C, and finally changed to 30 °C. The FCPIDNN controller successfully modulated the temperature of the modules to the setting targets and the square roots of the cost function got to minima at last.

**Figure 9:**
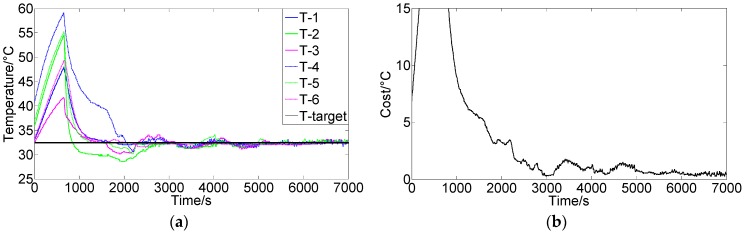
(**a**) Control results of the FCPIDNN controller when all fans were working (target temperature is 32.5 °C); (**b**) Variation of the square root of the cost function ($\sqrt{J(n)}$).

**Figure 10:**
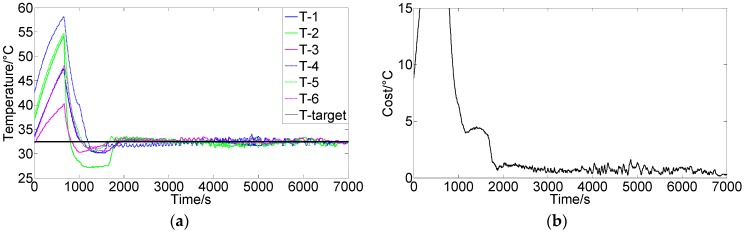
(**a**) Control results of the FCPIDNN controller when fan 2 failed (target temperature is 32.5 °C); (**b**) Variation of the square root of the cost function ($\sqrt{J(n)}$).

**Figure 11:**
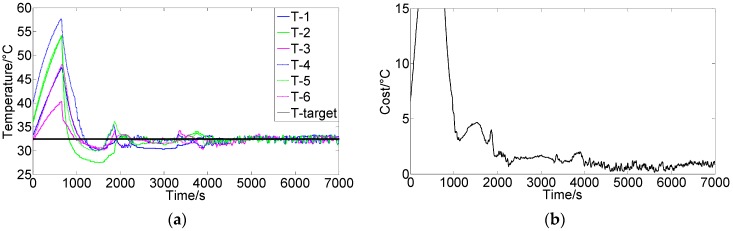
(**a**) Control results of the FCPIDNN controller when fan 3 failed (target temperature is 32.5 °C); (**b**) Variation of the square root of the cost function ($\sqrt{J(n)}$).

Reliability of temperature control is generally very important in practical use. When a fan fails to work, the system can make a self-reconfiguration to adapt to this situation. To validate the reliability, we conducted an experiment by setting the target temperature of all modules to 32.5 °C at the beginning of the process, and then shut off one fan. Figure 9a,b shows the control results when all fans were working. Figure 10 shows the control results when fan 2 failed. Figure 11 shows the control results when fan 3 failed. We can see that the temperatures of the modules could be modulated to the setting targets, even though one fan failed.

From the experiments, we can see that both FCPIDNN and PIDNN can control the system without the need of the model. The FCPIDNN can successfully deal with the coupling MIMO system with global optimum, but PIDNN can only control the temperature for one module, and will bring errors when controlling multiple coupling modules. In addition, FCPIDNN can make self-reconfiguration to the actuator fail. The above capabilities enhance the robustness of the control system and make the system smart, aiming at any possible scenario.

## 4. Conclusions

In this paper, we developed a MIMO self-tuning temperature sensing and control system with a novel FCPIDNN controller for an instrument with multi-modules. A mockup system was built up to mimic the complicated environment in the instrument and test the effectiveness of the MIMO self-tuning system. The MIMO temperature sensing and control system can adjust the internal temperature environment of the instrument without the need for system modeling. It can get to a global optimal result by on-line self-tuning. The system is applicable to effectively control the internal temperature of an instrument aiming at various mission scenarios, in particular, it is able to self-reconfigure upon actuator failure.

## References

[1] Zhang C.S., Xu J.L., Ma W.L., Zheng W.L. (2006). PCR microfluidic devices for DNA amplification. Biotechnol. Adv..

[2] Li C., Li Z., Jia H., Yan J. (2011). One-step ultrasensitive detection of microRNAs with loop-mediated isothermal amplification (LAMP). Chem. Commun..

[3] Yang S., Hsiung S., Hung Y., Chang C., Liao T., Lee G. (2006). A cell counting/sorting system incorporated with a microfabricated flow cytometer chip. Meas. Sci. Technol..

[4] Pires N.M.M., Dong T., Hanke U., Hoivik N. (2014). Recent Developments in Optical Detection Technologies in Lab-on-a-Chip Devices for Biosensing Applications. Sensors.

[5] Ahrberg C.D., Ilic B.R., Manz A., Neuzil P. (2016). Handheld real-time PCR device. Lab Chip.

[6] Joo S., Kim K.H., Kim H.C., Chung T.D. (2010). A portable microfluidic flow cytometer based on simultaneous detection of impedance and fluorescence. Biosens. Bioelectron..

[7] Roda A., Mirasoli M., Dolci L.S., Buragina A., Bonvicini F., Simoni P., Guardigli M. (2011). Portable Device Based on Chemiluminescence Lensless Imaging for Personalized Diagnostics through Multiplex Bioanalysis. Anal. Chem..

[8] Song Z., Murray B.T., Sammakia B. (2013). Airflow and temperature distribution optimization in data centers using artificial neural networks. Int. J. Heat Mass Transf..

[9] Shen Y., Cai W., Li S. (2010). Normalized decoupling control for high-dimensional MIMO processes for application in room temperature control HVAC systems. Control Eng. Pract..

[10] Pohjoranta A., Halinen M., Pennanen J., Kiviaho J. (2015). Model predictive control of the solid oxide fuel cell stack temperature with models based on experimental data. J. Power Sources.

[11] Moon U., Kim W. (2011). Temperature Control of Ultrasupercritical Once-through Boiler-turbine System Using Multi-input Multi-output Dynamic Matrix Control. J. Electr. Eng. Technol..

[12] Li J., Meng X. (2013). Temperature decoupling control of double-level air flow field dynamic vacuum system based on neural network and prediction principle. Eng. Appl. Artif. Intell..

[13] Gil P., Henriques J., Cardoso A., Carvalho P., Dourado A. (2014). Affine Neural Network-Based Predictive Control Applied to a Distributed Solar Collector Field. IEEE Trans. Control Syst. Technol..

[14] Shen L., He J., Yang C., Gui W., Xu H. (2016). Temperature Uniformity Control of Large-Scale Vertical Quench Furnaces for Aluminum Alloy Thermal Treatment. IEEE Trans. Control Syst. Technol..

[15] Lee C., Chen R. (2015). Optimal Self-Tuning PID Controller Based on Low Power Consumption for a Server Fan Cooling System. Sensors.

[16] Rossomando F.G., Soria C.M. (2015). Identification and control of nonlinear dynamics of a mobile robot in discrete time using an adaptive technique based on neural PID. Neural Comput. Appl..

[17] Maraba V.A., Kuzucuoglu A.E. (2011). PID Neural Network Based Speed Control of Asynchronous Motor Using Programmable Logic Controller. Adv. Electr. Comput. Eng..

[18] Stafford J., Walsh E., Egan V., Grimes R. (2010). Flat plate heat transfer with impinging axial fan flows. Int. J. Heat Mass Transf..

[19] Grimes R., Davies M. (2004). Air flow and heat transfer in fan cooled electronic systems. J. Electron. Packag..

